# HETERONORMATIVE SILENCES AND QUEER LONGINGS IN LGBTQ PEOPLE’S EXPERIENCES OF ELDER CARE AND HOME

**DOI:** 10.1093/geroni/igad104.2570

**Published:** 2023-12-21

**Authors:** Anna Siverskog

**Affiliations:** Södertörn University, Stockholm, Stockholms Lan, Sweden

## Abstract

The meaning of home for queer people have been widely empirically explored as well as theorized. Not least has the home been important for the older generations of queer people, who lived in times where their sexualities and gender identities have been criminalized, pathologized and where there have been few public meeting places historically. However, having care needs may blur the lines between private and public and complicate notions of integrity in one’s home. This paper is based on qualitative interviews and aims to explore experiences of LGBTQ people in a Swedish context who have eldercare services; either people who have home-care-services or who are living in care homes. A queer theoretical framework and thematic analysis was used. The results illustrate how there is a silence around gender and sexuality in the everyday life within eldercare. This in turn is caused by material conditions where downsizing and effectivization of the eldercare has created pressed working conditions that leaves little room for small talk between staff and recipients of care. Norms on age, gender, and sexuality with notions on older people as asexual (as well as cisgender and straight) may play into this silence as well. The boundaries between the private (home) and the public (eldercare) become blurred. This in turn conditions which intimacy practices that become im/possible. Simultaneously, there is a presence of queer resistance as well as of longings for other (queer) futures.

